# Supplementary figures and images for: Information integration and collective motility in phototactic cyanobacteria
Source: PLoS Comput Biol. 2020 Apr 30;16(4):e1007807. doi: 10.1371/journal.pcbi.1007807 (PMC7237038; doi:10.1371/journal.pcbi.1007807)

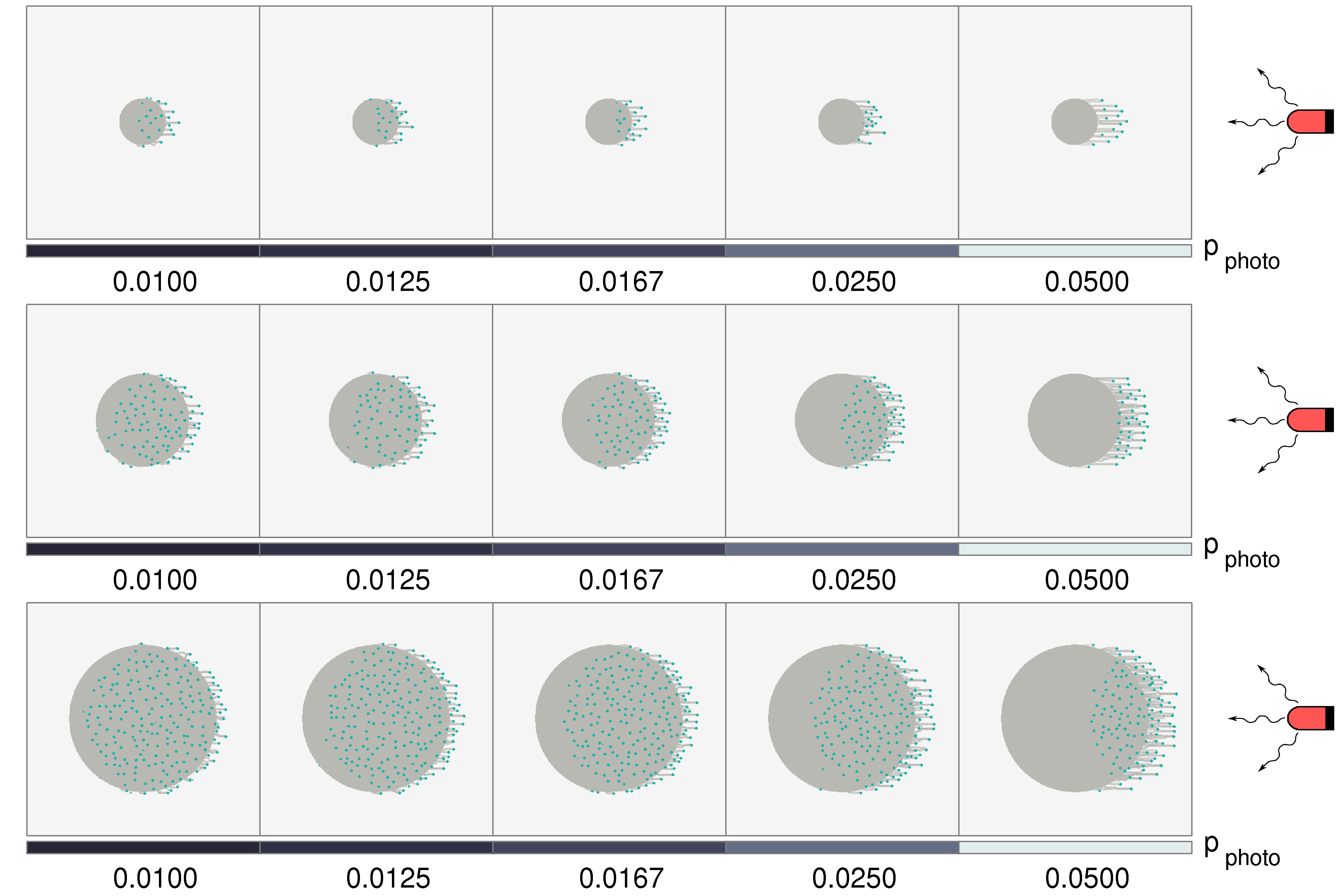

Supplement: S1 Fig — Colonies are illuminated by a single light source placed at the East. Each experiences a different intensity of incident light, depending on its location relative to the light source, such that the easternmost colony experiences the greatest incident light intensity. Thus, pphoto decreases linearly towards the West, as shown in the bar below each row. The number of cells in each row is indicated by N, and the model was simulated for 3 × 10^4^ time steps. Note that in each of the panels we have used a slime matrix of dimension 400 × 400, which is larger than those used for figures in the main text (300 × 300). (TIFF) [file pcbi.1007807.s001.tiff]

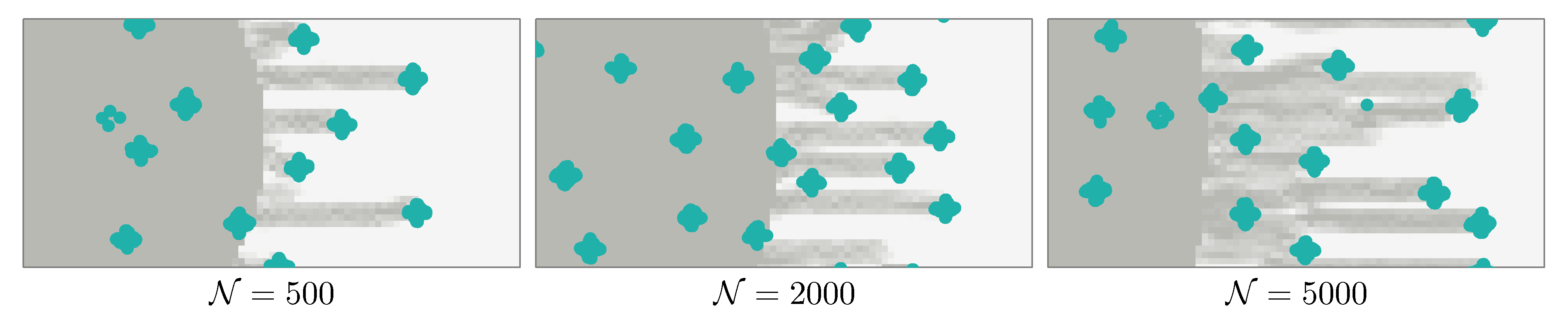

Supplement: S2 Fig — Morphologies of three colonies, each under illumination from a single light source placed at the East. The number of cells in each colony is (left-right) N=500, N=2000 and N=5000 cells, as indicated below the corresponding panels. Each colony experiences a light intensity of pphoto = 0.05 and the model was simulated for 3 × 10^4^ time steps. In each case, we display a close-up of the morphologies around the colony edge, where the dimensions of each box is identical. The displayed results indicate that the morphology of the fingers is relatively independent of the number of cells N. (TIFF) [file pcbi.1007807.s002.tiff]

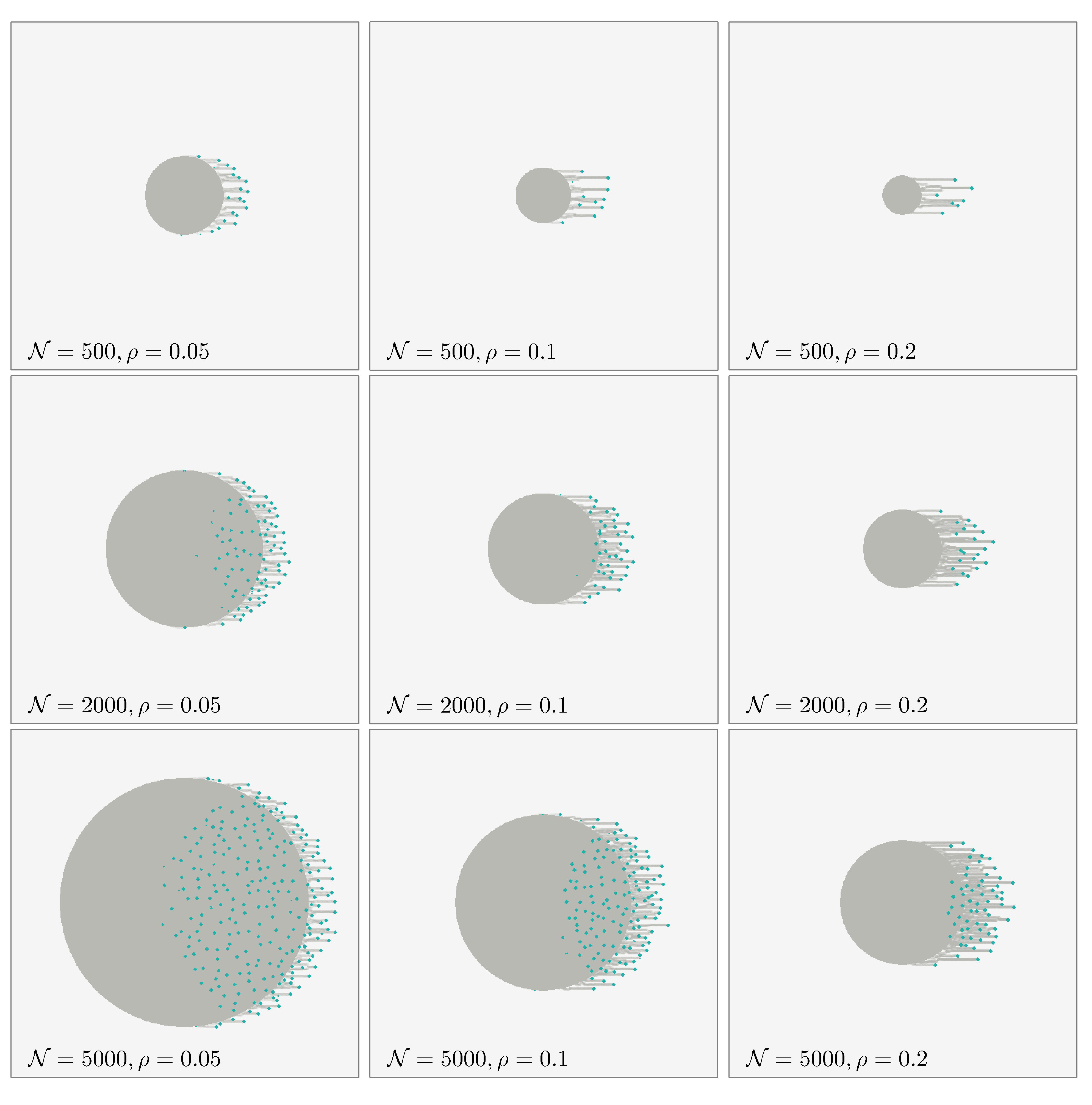

Supplement: S3 Fig — Morphologies of several colonies under illumination from a single light source placed at the East. Each colony experiences a light intensity of pphoto = 0.05, the number of cells in each panel is indicated by N, the colony density is indicated by ρ and the model was simulated for 3 × 10^4^ time steps. Note that in each of the panels we have used a slime matrix of dimension 500 × 500, which is larger than those used for figures in the main text (300 × 300). (TIFF) [file pcbi.1007807.s003.tiff]

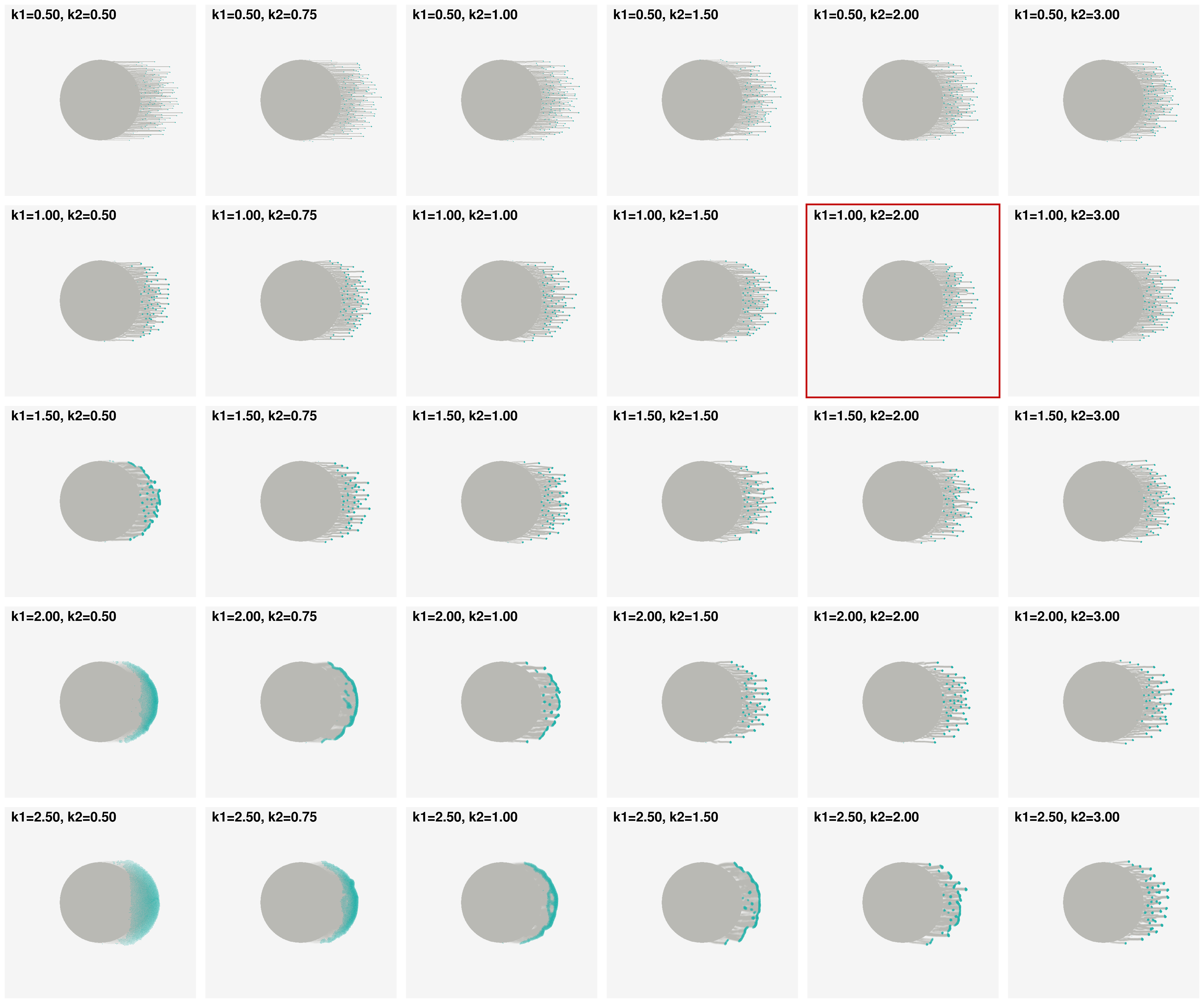

Supplement: S4 Fig — Snapshots of the colonies at t = 5 × 10^4^ for different values of the parameters k1 and k2 in a system of size N=5000 for the situation where a light source is placed at infinity (to the right of the colony). The panel highlighted by the red box shows results obtained using k1 = 1 and k2 = 2, which are the values used in the manuscript. (TIFF) [file pcbi.1007807.s004.tiff]

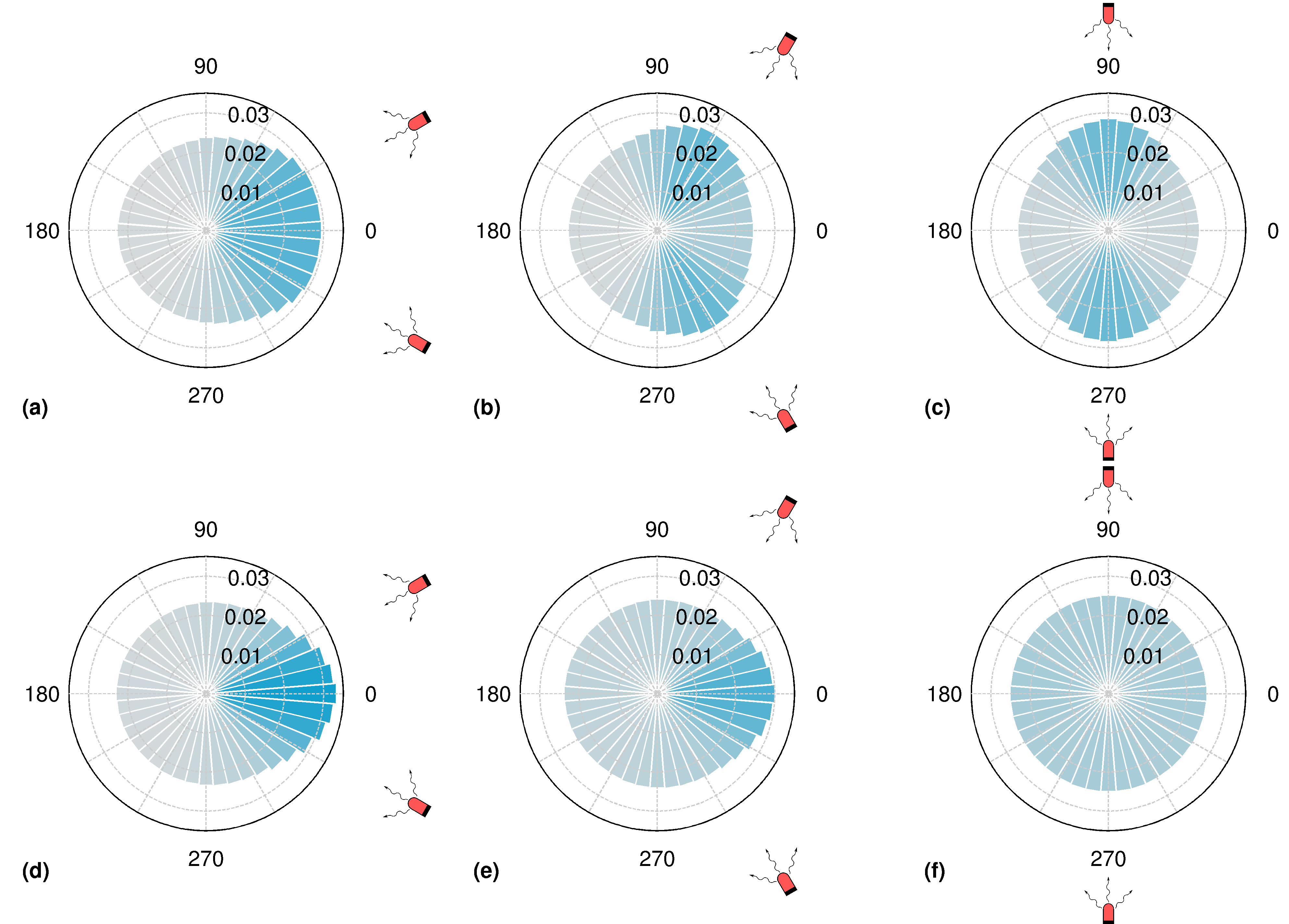

Supplement: S5 Fig — Distribution of angles of motion of cells in colonies exposed to a pair of light sources placed at different positions relative to it. The probabilities are represented by the histogram of the angles made by the movement of a cell in a single time step. The light sources are placed at angles 30° (a,d), 60° (b,e) and 90° (c,f) North/South of East as shown in the respective figures. In (a-c) at each step cells stochastically bias their motion in the direction of one of the light sources. In (d-f) at each step cells bias their motion towards the vector sum of the light sources. In all cases, the intensity of color of the bars of the rose plots are related to their magnitude. Each colony contains N=5000 cells, and the model was simulated for 3 × 10^4^ time steps for the case p1 = p2 = 0.05. (TIFF) [file pcbi.1007807.s005.tiff]

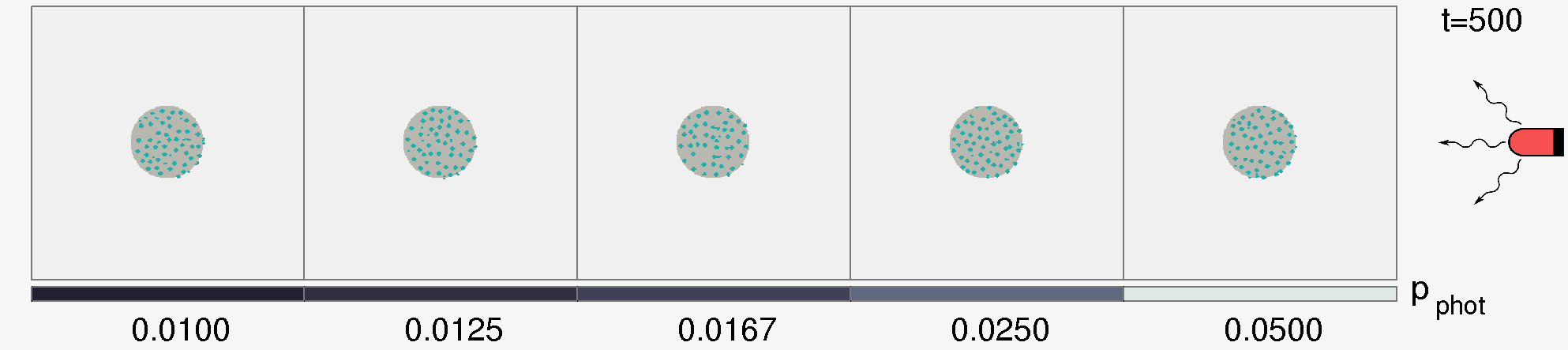

Supplement: S1 Movie — We start with an array of colonies under a single light source placed to the East of the colonies as described in Fig. 3. Here pphoto decreases linearly towards the West, as shown in the bottom bar. Each subsequent frame is separated by 500 time steps. Each colony contains N = 500 cells, and the model was simulated for 4 × 10^4^ time steps. (GIF) [file pcbi.1007807.s006.gif]
